# Genetic architecture for skeletal muscle glycolytic potential in Chinese Erhualian pigs revealed by a genome-wide association study using 1.4M SNP array

**DOI:** 10.3389/fgene.2023.1141411

**Published:** 2023-03-17

**Authors:** Xinke Xie, Cong Huang, Yizhong Huang, Xiaoxiao Zou, Runxin Zhou, Huashui Ai, Lusheng Huang, Junwu Ma

**Affiliations:** ^1^ National Key Laboratory for Swine Genetic Improvement and Production Technology, Ministry of Science and Technology of China, Jiangxi Agricultural University, Nanchang, China

**Keywords:** Erhualian, pig, glycolysis potential, GWAS, candidate gene

## Abstract

**Introduction:** Muscle glycolytic potential (GP) is a key factor affecting multiple meat quality traits. It is calculated based on the contents of residual glycogen and glucose (RG), glucose-6-phosphate (G6P), and lactate (LAT) contents in muscle. However, the genetic mechanism of glycolytic metabolism in skeletal muscle of pigs remains poorly understood. With a history of more than 400 years and some unique characteristics, the Erhualian pig is called the “giant panda” (very precious) in the world’s pig species by Chinese animal husbandry.

**Methods:** Here, we performed a genome-wide association study (GWAS) using 1.4M single nucleotide polymorphisms (SNPs) chips for longissimus RG, G6P, LAT, and GP levels in 301 purebred Erhualian pigs.

**Results:** We found that the average GP value of Erhualian was unusually low (68.09 μmol/g), but the variation was large (10.4–112.7 μmol/g). The SNP-based heritability estimates for the four traits ranged from 0.16–0.32. In total, our GWAS revealed 31 quantitative trait loci (QTLs), including eight for RG, nine for G6P, nine for LAT, five for GP. Of these loci, eight were genome-wide significant (*p* < 3.8 × 10^−7^), and six loci were common to two or three traits. Multiple promising candidate genes such as *FTO*, *MINPP1*, *RIPOR2*, *SCL8A3*, *LIFR* and *SRGAP1* were identified. The genotype combinations of the five GP-associated SNPs also showed significant effect on other meat quality traits.

**Discussion:** These results not only provide insights into the genetic architecture of GP related traits in Erhualian, but also are useful for pig breeding programs involving this breed.

## Introduction

Meat quality significantly influences consumers’ preference and purchasing behavior, thus improving meat quality has been being a major goal of pig breeding (Khanal et al., 2019; Zhang et al., 2019). In recent decades, the meat quality of commercial lean pigs has been improved to a certain extent by removing some known unfavorable alleles for meat quality such as PRKAG3 (200Q) (Milan et al., 2000) and RYR1 (615C) alleles (Fujii et al., 1991); however, their meat quality is still cannot meet the demands of discerning consumers (Bonneau and Lebret, 2010). A prominent problem of meat quality of commercial pigs is the high incidence (10%–30%) of inferior meat, including pale, soft, exudative (PSE) meat and dark, firm, dry (DFD) meat, which causes huge economic losses to pig industry and pork processing industry (Lewis et al., 1987; Van der Wal et al., 1988; Shen et al., 2006; Gajana et al., 2013; Gonzalez-Rivas et al., 2020). In contrast, PSE and DFD meat are rarely found in Chinese native pigs, making them valuable genetic resources for cultivating high-quality pig synthetic lines (Chen et al., 2013; Ma et al., 2013; Liu et al., 2015; Wu et al., 2020).

The degree of post-mortem pH decline in porcine skeletal muscle is an important factor accounting for the occurrence of inferior meat, as it significantly affects meat color development, water-holding capacity and tenderness (Bee et al., 2007; Scheffler and Gerrard, 2007; Popp et al., 2015). The ultimate pH value of PSE and DFD meat is usually lower than 5.5 and greater than 6.0, respectively. It is well known that muscle pH value and other pork quality attributes depend on the content of glycogen of muscle at slaughter, as well as the rate and extent of lactate accumulation during 24 h post-slaughter glycolysis metabolism (Sayre et al., 1963; Hamilton et al., 2003). Based on that, muscle glycolytic potential (GP), defined as the potential of lactate production during post-mortem glycolysis, has been proposed as a predictor for the extent of pH decline (Maribo et al., 1999; Scheffler and Gerrard, 2007).

To date, two major genes have been identified to strongly influence GP phenotype and other meat quality traits in commercial pigs. *PRKAG3* is the first causal gene proved to affect GP level, and its R200Q mutation significantly increases muscle glycogen content by up to ∼70%, consequently causing acidic meat in Hampshire pigs (Milan et al., 2000). In addition, it was found in Duroc and its hybrid pigs that a splice mutation (g.8283C > A) in the *PHKG1* gene caused a 43% increase in GP and a 20% decrease in drip loss of pork (Ma et al., 2014). These two gene mutations with large effects on GP-related traits are all derived from European commercial pig breeds but are rarely present in Chinese native pigs. However, few studies have systematically investigated the genetic architecture of GP-related traits in Chinese native pigs.

The Chinese Erhualian pig is famous for its high prolificacy (Li et al., 2017). Its meat quality is also good and superior to European commercial pig breeds in color, pH value, water-holding capacity, marbling, intramuscular fat, tenderness, muscle fiber diameter, taste and flavor (Chen et al., 2013). Intriguingly, our recent study demonstrated that compared with Chinese Bama Xiang and Laiwu pigs, Erhualian pigs had a higher muscle ultimate pH value (pH_u_), a smaller extent of pH decline (pH_d_) from post-mortem 45 min to 24 h, and a greater variation in the two pH index (Huang et al., 2020). The variation in muscle pH value of Erhualian may be at least partially due to the variation in GP level or the concentrations of its components, including residual glycogen and glucose (RG), glucose-6-phosphate (G6P) and lactate (LAT). To verify this speculation, we herein first assessed the variation of GP-related traits in 301 Erhualian pigs, and then evaluated the impact of GP on pH values. Furthermore, to reveal the genetic architecture of the GP phenotypes of Erhualian pigs, we performed a genome-wide association study (GWAS) using high-density 1.4M single nucleotide polymorphisms (SNPs) chip data from the population.

## Materials and methods

### Animals and sampling

The Erhualian population was established as described previously (Liu et al., 2015). Briefly, the population consists of 160 barrows (castrated males) and 141 gilts, which were produced from 11 boars and 51 dams. These piglets were all born in Jiaoxi and transferred to Nanchang when they were 2–3 months old. Then they were raised under uniform conditions and slaughtered in 11 batches in the same commercial abattoir when they were about 300 days old. At post-mortem 30 min, 2–3 g muscle samples were collected from the *longissimus thoracis* (LT) and then stored at −80°C until assay.

### Phenotype measurement and correlation analysis

The contents of residual glycogen and glucose (RG), glucose-6-phosphate (G6P) and lactate (LAT) in LT muscle were determined using the glycogen assay kits (E2GN-100) BioAssay Systems, the glucose-6-phosphate assay kits (EG6P-100) BioAssay Systems and the lactic acid assay kits (A019-2) from the Nanjing Jiancheng Bioengineering Institute, respectively. GP were calculated as the sum of: 2 × (RG + G6P) + Lactate (Monin and Sellier, 1985) and expressed as μmol of lactic acid equivalent per g of fresh muscle. In addition, pH values of LT muscle was measured twice on each sample at 45 min (pH_i_) and 24 h (pH_u_) after slaughter using a Delta 320 pH meter, and their mean values were calculated separately. The difference between pH_i_ and pH_u_ was taken as pH decline (pH_d_). Drip loss was assayed using an EZ-Drip Loss method. Three color parameters L^*^, a^*^ and b^*^ on the surface cuts of LT were objectively evaluated with a CM-2600d/2500d Minolta Chroma meter (Liu et al., 2015). The correlations between the GP related traits and the pH traits were evaluated using Pearson correlation analysis.

### Genotyping and quality control

Genomic DNA was extracted from pig ear tissue using standard phenol/chloroform extraction. In order to meet the genotyping requirements, all DNA samples were standardized to a final concentration of 50 ng/μL and quality controlled. Totally, 301 Erhualian pigs were genotyped using the 1.4M SNP Beadchips, which is a customized Affymetrix Axiom chip. The SNPs on the chip were found from whole genome sequence data of 150 Chinese indigenous pigs and 38 International commercial pigs, and were evenly distributed across the pig genome (Gong et al., 2019). PLINK v1.9 was used for quality control of the SNP chip data (Purcell et al., 2007). SNPs with a call rate ≥ 0.90 or a minor allele frequency (MAF) ≥ 0.05, and samples with a call rate greater than 95%, were retained. Consequently, 732,609 SNPs and all individuals were kept for GWAS analysis.

### Genome-wide association analyses for GP related traits

We used linear regression models to adjust GP related traits for sex and slaughter batch, and then applied Genome-wide Efficient Mixed-Model Association (GEMMA) method for genetic association analyses and SNP-based heritability estimation, which were conducted using the “−lmm 1” and “−gk 1” commands of GEMMA software (Zhou and Stephens, 2012). GEMMA examined the associations of SNPs with phenotypic values under the following linear mixed model:

Y=Sa+u+e

where **Y** is a vector of residual phenotypic values that were corrected for fixed effects (sex and slaughter batch) using lm function in R program. **S** is the incidence vector for **a**, and **a** is the additive genetic effect of the SNP under test; **u** is a vector of random polygenic effects that is assumed to follow a multivariate normal distribution MVN(0, **G** $\sigma_{a}^{2}$ ), where **G** is genomic relationship matrix that was constructed based on qualified SNPs and $\sigma_{a}^{2}$ is the polygenetic additive variance; **e** is a vector of residual errors with a distribution of N (0, **I** $\sigma_{e}^{2}$ ), where **I** is identity matrix and $\sigma_{e}^{2}$ is residual variance.

Bonferroni correction method was used to set the genome-wide significant (0.05/N) and suggestive (1/N) association thresholds, where N is the number of independent association tests or SNPs (Lander and Kruglyak, 1995; Yang et al., 2005). Considering that SNP clusters in high linkage disequilibrium (LD) may cause overestimate of N and significance thresholds, we first pruned the full SNP set (732,609 SNPs that passed quality control) to 130,404 independent SNPs (*r* ^2^ < 0.3) by the command “indep-pairwise 50 5 0.3” in PLINK v1.9. Therefore, a SNP was considered to be genome-wide significance at *p* < 3.8 × 10^−7^ (0.05/130,404), and to be suggestive significance at *p* < 7.6 × 10^−6^ (1/130,404). The impact of population stratification was estimated by the quantile-quantile (Q-Q) plot and genotype data PCA analysis (Pearson and Manolio, 2008). The phenotypic variances explained by the significant SNPs were estimated by (V_reduce_ - V_full_)/V_reduce_, where V_full_ and V_reduce_ are residual variances of models for association analysis with and without the SNP term, respectively. Haplotype block or LD analysis was performed for the chromosomal regions with multiple significant SNPs clustered around the peak SNP. Haplotype blocks were identified using the HAPLOVIEW v4.2 software with default settings (Barrett et al., 2005).

## Results

### Descriptive and correlation statistics of GP related traits

The descriptive statistical results of four GP-related traits (RG, G6P, LAT and GP) in the longissimus muscle of 301 Erhualian pigs were given in Table 1. Our data showed that the average value of GP in Erhualian was 68.09 μmol/g (Table 1). The coefficient of variation of GP related traits was relatively large (0.30–0.55). Moreover, these traits had low to moderate heritability (0.16–0.32).

**TABLE 1:** Descriptive statistics of four muscle GP-related traits in 301 Erhualian pigs.

Trait	Mean ± SD^a^ (µmol/g)	Max. (µmol/g)	Min. (µmol/g)	CV^b^	*h* ^2^ (se)^c^
RG	6.88 ± 3.78	16.99	0.16	0.55	0.20 (0.11)
G6P	5.46 ± 2.59	15.44	0.32	0.47	0.32 (0.13)
LAT	43.41 ± 13.15	76.40	6.27	0.30	0.16 (0.09)
GP	68.09 ± 21.30	112.71	10.42	0.31	0.24 (0.10)

^a^ Standard deviation.

^b^ Coefficient of variation.

^c^ Heritability estimates (standard errors).

**FIGURE 1:**
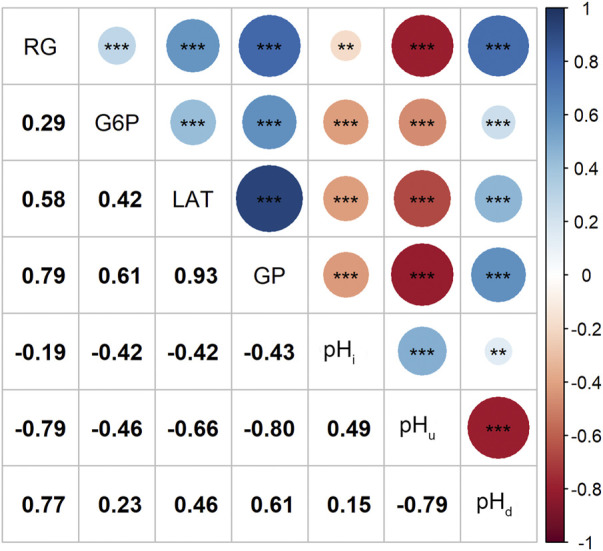
Magnitudes of correlations between the four GP related traits and the 3 pH related traits, ^**^: *p* < 0.01, ^***^: *p* < 0.001.

Correlation analysis results showed that the contents of RG, G6P and LAT were not only positively correlated with each other, but also had a strong and positive correlation with the GP content (r ≥ 0.61, *p* < 0.001; Figure 1). Since changes in muscle glucose metabolism may lead to changes in its pH values, we estimated the correlations between the four GP-related traits and pH index. As expected, our data indicates that higher RG and GP contents tended to cause a greater post-mortem pH decline (pH_d_) and a lower ultimate pH (pH_u_) value (r ≤ −0.79, *p* < 0.001; Figure 1), which is consistent with other studies (Hamilton et al., 2003; Luo et al., 2017).

### Summary of GWAS results

**FIGURE 2:**
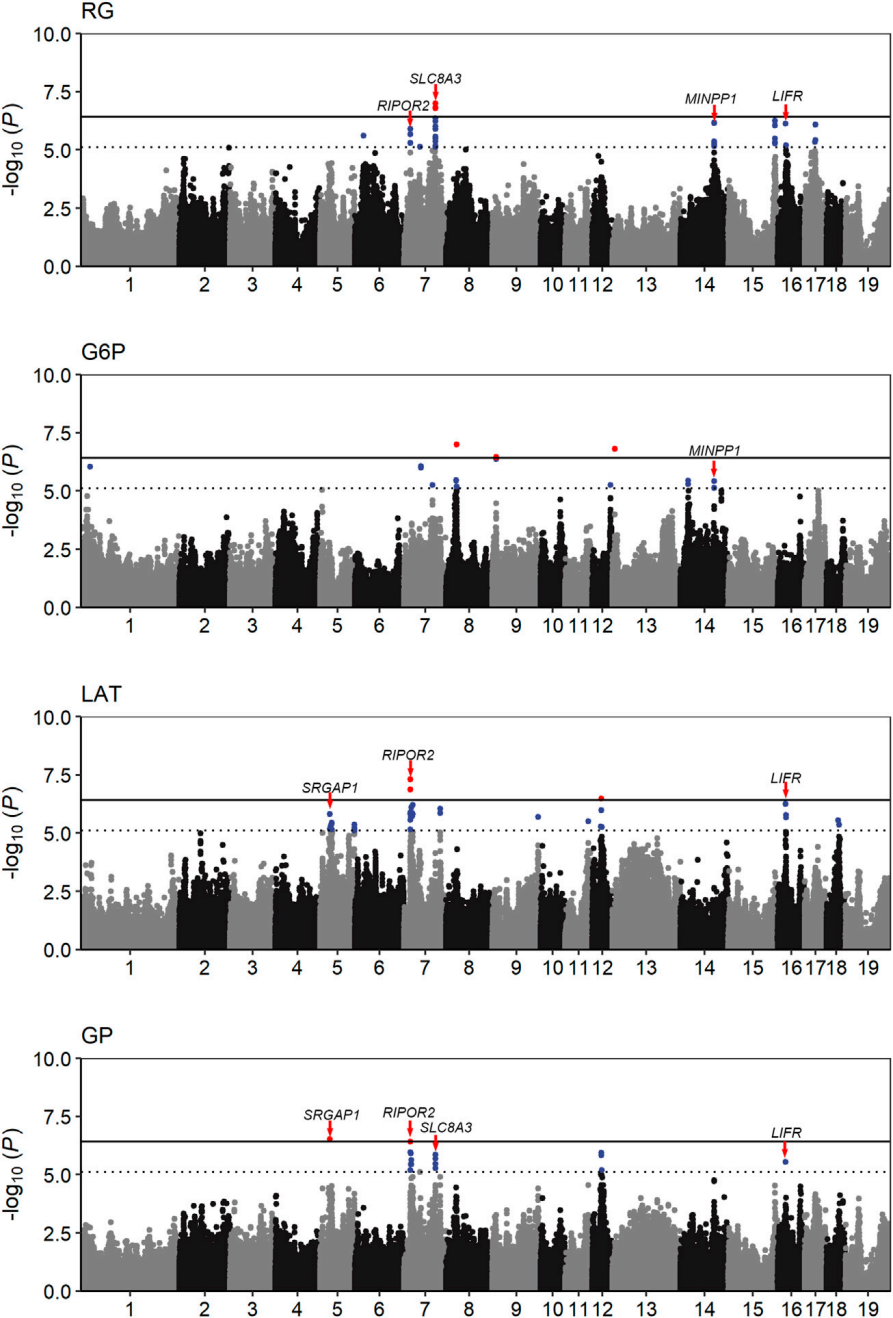
Manhattan plots of the GWAS result for the four GP related traits. The solid and dotted lines represent the genome-wide significance threshold and the suggestive significance threshold, respectively. The candidate genes close to five pleiotropic loci were identified.

The population stratification was evaluated using Q-Q plots and PCA plots (Supplementary Figure S1, S2). The inflation factors (λ) for four traits were between 1.018 and 1.119 and PCA analysis showed that the population was relatively clustered, which suggests that our population had no obvious stratification. Through GWAS analysis, we totally identified 31 quantitative trait loci (QTLs) for the four GP related traits (Table 2; Figure 2), including eight for RG, nine for G6P, nine for LAT, five for GP. Notably, six out of the 31 QTLs showed significant association with more than one trait, including the QTLs with lead SNPs rs331183308 for RG and G6P, rs321246758 for RG and GP, rs318410870 and rs318442172 for RG, LAT and GP, and rs697205060 and rs345106152 for LAT and GP. Perhaps because LAT content accounted for the major fraction of GP at 45 min post-mortem (43.41/68.09 ≈ 63.8%; Table 1), they shared four common GWAS peak SNPs (Table 2).

**TABLE 2:** The summary of top SNPs significantly associated with muscle GP related traits.

Trait	Peak SNP^1^	Pleiotropic^2^	Chr^3^	Pos (bp)^4^	Freq^5^	Beta (s.e)^6^	*p*-value^7^	Nearest gene	Location	Var(%)^8^
RG	rs338058884		6	31,271,210	0.102	−2.02 (0.42)	2.15E-06	*FTO*	intron	7.8
RG	rs318410870	2	7	19,677,069	0.246	−1.43 (0.29)	1.25E-06	*RIPOR2*	intron	5.3
RG	rs80841859		7	44,653,377	0.463	−1.24 (0.27)	7.49E-06	*TFAP2D*	intron	9.2
RG	rs321246758	3	7	93,785,804	0.423	−1.45 (0.27)	9.86E-08	*SLC8A3*	intron	8.7
RG	rs331183308	6	14	99,636,228	0.322	1.48 (0.29)	6.67E-07	*MINPP1*	intergenic	3.6
RG	15_135271601		15	135,271,601	0.417	1.43 (0.28)	5.68E-07	*AGAP1*	intergenic	5.2
RG	rs318442172	5	16	23,986,799	0.331	1.34 (0.27)	7.58E-07	*LIFR*	intergenic	6.7
RG	rs322341359		17	29,911,510	0.083	2.4 (0.48)	8.02E-07	*FOXA2*	intergenic	7.1
G6P	rs323123457		1	25,796,643	0.355	0.93 (0.19)	9.15E-07	*NHSL1*	intron	13.1
G6P	rs319985393		7	48,002,599	0.457	0.92 (0.18)	8.63E-07	*ADAMTS7*	intergenic	9
G6P	rs326947848		7	85,861,162	0.5	1.04 (0.22)	5.56E-06	*RGMA*	intergenic	6.3
G6P	rs320783325		8	32,614,482	0.257	1.14 (0.21)	9.81E-08	*LIMCH1*	intron	10.2
G6P	rs330779127		9	11,154,160	0.434	−0.93 (0.18)	3.43E-07	*ACER3*	intron	12.4
G6P	rs325508440		12	55,011,608	0.231	−1.08 (0.23)	5.63E-06	*MYH13*	intergenic	2.4
G6P	rs333704759		13	7,126,930	0.47	−0.96 (0.18)	1.56E-07	*SGO1*	intergenic	11
G6P	rs340666100		14	23,234,922	0.338	1.08 (0.23)	3.63E-06	*GALNT9*	Downstream gene	8.6
G6P	rs337801210	6	14	99,543,552	0.114	1.49 (0.32)	3.70E-06	*MINPP1*	intergenic	8.6
LAT	rs697205060	1	5	28,321,339	0.127	6.78 (1.38)	1.49E-06	*SRGAP1*	intron	8.8
LAT	rs80827576		5	31,833,661	0.15	6.2 (1.33)	4.44E-06	*CAND1*	intergenic	8.5
LAT	rs318410870	2	7	19,677,069	0.246	−6.12 (1.09)	4.86E-08	*RIPOR2*	intron	8.6
LAT	rs332736034		7	109,115,167	0.279	−5.84 (1.17)	9.16E-07	*ENSSSCG00000042684*	intergenic	9
LAT	rs327466581		9	133,730,734	0.111	7.59 (1.57)	2.03E-06	*ENSSSCG00000015620*	intergenic	6.6
LAT	rs345209200		11	67,518,507	0.491	−4.84 (1.02)	3.14E-06	*STK24*	intron	9.7
LAT	rs345106152	4	12	28,646,375	0.434	−4.83 (0.92)	3.27E-07	*ENSSSCG00000043336*	upstream	10
LAT	rs318442172	5	16	23,986,799	0.331	5.06 (0.99)	5.64E-07	*LIFR*	intergenic	8.6
LAT	rs330527025		18	34,087,184	0.459	4.61 (0.97)	2.83E-06	*IMMP2L*	intron	5.3
GP	rs697205060	1	5	28,321,339	0.127	11.23 (2.14)	3.06E-07	*SRGAP1*	intron	5.6
GP	rs318410870	2	7	19,677,069	0.246	−9.40 (1.81)	3.77E-07	*RIPOR2*	intron	8.8
GP	rs332409349	3	7	93,944,227	0.326	−8.95 (1.82)	1.40E-06	*SYNJ2BP*, COX16	intron	5.6
GP	rs345106152	4	12	28,646,375	0.434	−7.58 (1.53)	1.14E-06	*ENSSSCG00000043336*	upstream gene	12.2
GP	rs318442172	5	16	23,986,799	0.331	7.93 (1.66)	2.85E-06	*LIFR*	intergenic	9.7

^1^One SNP, wthout feature ID (rs) in NCBI, was named according to their physical positions on the Sscrofa11.1 assembly.

^2^The numbes indicated pleiotropic loci associated with more than one trait.

4 3The locations of the associated SNPs, on the *Sus Scrofa* Build 11.1 assembly.

^5^Minor allele frequeny.

^6^Beta estimates (stanard errors for beta).

^7^The *p* values that ar lower than the genome-wide significance threshold (3.8 × 10^−7^) are underlined.

^8^Phenotypic variance hat the peak SNP, explain.

### QTLs for RG trait

The RG level in muscle at post-mortem 45 min is weakly associated with pH_i_ (r = −0.19, *p* < 0.01) but strongly associated with pH_d_ (r = 0.77, *p* < 0.01; Figure 1), suggesting that it plays an important role in promoting the subsequent decline of pH in meat. In the Erhualian pigs, a total of eight QTLs associated with RG were identified, which individually explained 3.6%–9.2% of the phenotypic variance. Among them, only the QTL with peak SNP rs321246758 (*p* = 9.86 × 10^−8^), which is located in the intron of *SLC8A3* gene on *Sus scrofa* chromosome 7 (SSC7), exceeded the genome-wide significance threthold (Table 2). This QTL region for RG overlapped with the QTL region for GP, in which the peak SNPs rs321246758 for RG and the peak SNP rs332409349 for GP was in moderate LD (*r* ^2^ = 0.63) (Supplementary Figure S3A), and a common SNP rs322439157 significantly associated with both traits was found, suggesting the existence of a pleiotropic locus in the *SLC8A3*–*SYNJ2BP* gene region (93.78–93.95 Mb) on SSC7 (Supplementary Figure S3C). The minor allele of rs322439157 were correlated with decreased levels of both RG and GP (Supplementary Figure S3B). In addition to rs321246758, three other GWAS signals appeared in the genes’ introns, including rs338058884, rs80841859, and rs318410870 located in the introns of *FTO*, *TFAP2D* and *RIPOR2*, respectively.

### QTLs for G6P

The conversion of glucose to G6P is the first rate-limiting step in the glycolysis pathway. We detected nine QTLs significantly associated with G6P, including three genome-wide significant QTLs (*p* < 3.8 × 10^−7^) with the peak SNPs rs320783325, rs330779127 and rs333704759 on SSC8, 9 and 13, respectively. Among them, the rs320783325, an intron variant in *LIMCH1* gene, had the most significant association with G6P (*p* = 9.81 × 10^−8^). In addition, we found that on SSC14, the GWAS peak SNP rs337801210 for G6P was located 92.7 kb proximal to the GWAS peak SNP rs331183308 for RG (Table 2). Although a weak LD (*r* ^2^ = 0.21) were observed between rs337801210 and rs331183308, they and nine other significant SNPs (2 for G6P and seven for RG) linked to them were concentrated in the 99.52–99.64 Mb region, which only contains the *MINPP1* gene. Moreover, the minor alleles of rs337801210 and rs331183308 were both associated with increased phenotypic values (Table 2). Thus, there may be a pleiotropic QTL in the *MINPP1* region that affects both G6P and RG.

### QTLs for LAT

Under anaerobic conditions, the end product of glycolysis in muscle cells is lactic acid. Our GWAS also revealed nine QTLs significantly associated with LAT (Table 2; Figure 2). Of them, two reached genome-wide significance: one on SSC7 with peak SNP rs318410870 (*p* = 4.86 × 10^−8^), and another on SSC12 with the peak SNP rs345106152 (*p* = 3.27 × 10^−7^). Moreover, these two peak SNPs were also significantly with GP. Notably, the pleiotropic locus rs345106152 explained the largest portion of phenotypic variance for LAT (10%) and GP (12.2%) (Table 2).

### QTLs for GP

**FIGURE 3:**
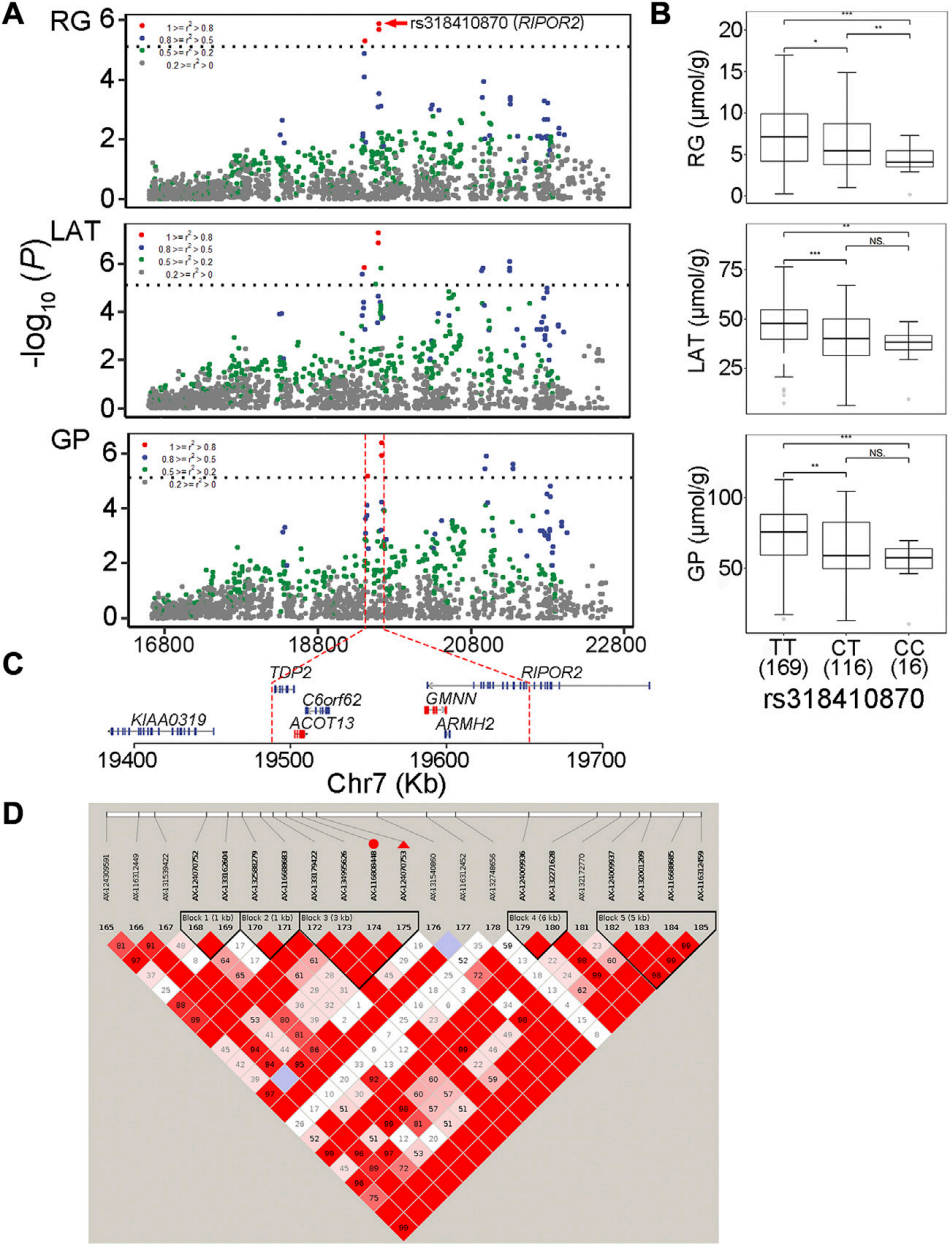
The GWAS peak SNP rs318410870 in RIPOR2 gene with pleiotropic effect on residual glycogen and glucose (RG), lactate (LAT) and glycolytic potential (GP) levels in muscle **(A)** Regional association plots of the QTL region centered by rs318410870 (in red dots). Different colors stand for degrees of linkage disequilibrium (r2) between corresponding SNPs and the peak SNP **(B)** Boxplots showing the difference in phenotypic values between the three genotypes of the SNP rs318410870 **(C)** The physical locations of genes surrounding the significant SNPs **(D)** Haplotype blocks on the QTL region containing rs318410870 indicated with red triangle and its one neighboring significant SNP indicated with red dot associated with the three traits.

In this study, two genome-wide significant QTLs and three suggestive QTLs were found to affect the GP trait. These five QTLs for GP were also significantly associated with at least one of the three GP components (RG, G6P and LAT). The GWAS peak SNPs rs697205060 (*p* = 3.06 × 10^−7^) and rs318410870 (*p* = 3.77 × 10^−7^) represented the genome-wide significant QTLs on SSC5 and SSC7, respectively. For rs697205060, an intron variant of *SRGAP1*, its minor allele was associated with increased concentrations of GP and LAT, while the minor allele at rs318410870, an intron variant of the *RIPOR2* gene, was associated with reduced concentrations of GP, RG and LAT (Table 2; Figures 3A–C). Furthermore, the LD analysis showed that the peak SNP rs318410870 and other three SNPs (including the second significant SNP) were all located in a 3-kb LD block in the *RIPOR2* gene (Figure 3D). In addition, on SSC16, the SNP rs318442172 near the *LIFR* gene was also detected to be significantly associated with GP, RG and LAT.

### Effects of multiple pleiotropic loci combination on meat quality traits

**FIGURE 4:**
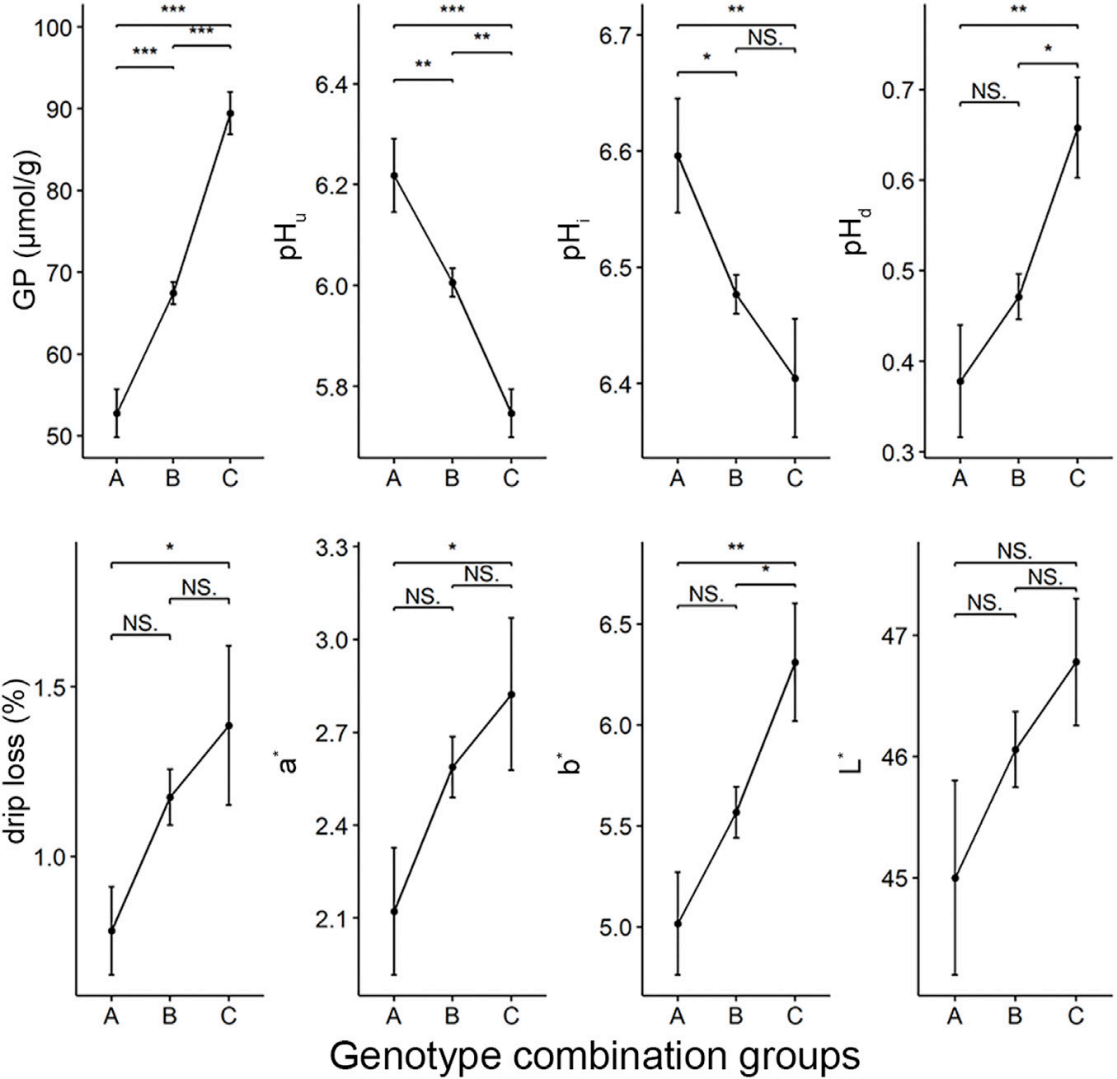
The differences in meat quality traits between three groups of genotype combinations of five GWAS peak SNPs for GP. The color parameters L^*^, a^*^, b^*^ indicate the lightness, redness and yellowness of the meat, respectively. There were 31, 234 and 30 individuals in group A, B and C respectively.

Next, we asked what would happen to other meat quality traits if we selected the five loci that affect GP and other components (including rs697205060, rs332409349, rs318410870, rs345106152, and rs318442172) in breeding. To this end, we first identified the genome combination types of these five SNPs in the Erhualian population. 73 genotype combinations were found in this population (Supplementary Table S1). According to the number of alleles (called allele_2_) that increase GP values at the five peak SNPs in each individual’s genotype combination, we divided all individuals into three groups: group A with 0–2 alleles_2_, group B with three to seven alleles_2_, and group C with 8–10 alleles_2_. Analysis of variance showed that there were significant differences in GP, pH_i_, pH_u_, pH_d_, drip loss, redness (a^*^) and yellowness (b^*^) of meat between the three groups, but no significant differences in lightness (L^*^). Moreover, the GP, pH_d_, drip loss, a^*^ and b^*^ values tended to increase in the genotype combination groups with more allele_2_, while their corresponding pH_i_ and pH_u_ showed a significant downward trend (Figure 4).

## Discussion

Many studies have investigated the variation of muscle GP level and its correlation with meat quality in Western commercial pig purebreds, crossbreds and Western Chinese hybrid pigs (van Laack and Kauffman, 1999; Bee et al., 2006; Duan et al., 2009; Conte et al., 2021), but such studies have been rarely conducted in Chinese indigenous pig breeds. In this study, the post-mortem muscle glycolytic phenotype of Erhualian pigs was characterized for the first time. We found that the average GP value of Erhualian was 68.09 μmol/g, which is only nearly half of the values reported by D’Astous-page et al. (D'Astous-Pagé et al., 2017) for Duroc (161.06 μmol/g), Landrace (164.27 μmol/g) and Yorkshire (159.27 μmol/g) and the value reported by Duan et al. (Duan et al., 2009) for a White Duroc × Erhualian F2 pigs (136.66 μmol/g). In addition, we showed that the RG and GP levels were highly negatively (r ≤ −0.79, *p* < 0.001) correlated with pH_u_, which is consistent with the notion that higher glycogen or GP at slaughter leads to greater pH_u_ decline (Scheffler and Gerrard, 2007). Given the significant effect of GP on pH decline in muscle, it can be inferred that the lower glycogen and glucose contents in the muscle of Erhualian before slaughter may be the main reason why its meat pH_u_ value was higher than that of Western commercial pig breeds (6.01 vs. ∼5.5) (Huang et al., 2020). In addition, the four GP-related traits of the Erhualian pigs showed considerable variation (CV = 30%–55%; Table 1), which may largely explain the wide variation of some meat quality traits related to GP in this population, such as the CV (63.4%) of pH_d_ value (Huang et al., 2020).

Numerous QTLs for meat quality traits have been identified in various F2 pig crosses between Chinese and Western outbred lines or in Western commercial hybrid pigs, but few QTLs have been detected in founder lines, which to some extent hinder the development of pure-breeding and cross-breeding. To our best knowledge, this is the first study to explore genetic loci for GP-related traits in a Chinese local pig breed (Erhualian). Unlike most pig GWAS using 50 K or 60 K SNP chips, this study used 1.4 M SNP chips, which improved the statistical power and QTL mapping resolution. In fact, we not only detected 31 QTLs (8 loci per trait on average), but also found QTLs in some regions with small LD.

By comparing with the QTLs for GP deposited in pig QTL database (pigQTLdb), we found that all QTLs detected in this study, except for two, had not been previously reported. On SSC7, the GWAS peak locus (rs332736034) we identified for LAT at 109.12 Mb is close to the QTL for the same trait detected in a Meishan × Pietrain F2 family (Reiner et al., 2002). Similarly, on SSC15, the significant region associated with RG of Erhualian overlapped with the QTL interval found in a Berkshire × Yorkshire F2 intercross (Ciobanu et al., 2001). Thus, the alleles at the two loci may be segregating rather than fixed in Erhualian pigs.

In the regions around the GWAS peaks, we screened a number of candidate genes, providing new clues for understanding the genetic mechanism of glycolytic phenotypes. For the identified pleiotropic loci, five strong candidate genes were proposed based on their functional annotations. The GWAS signal (rs331183308) for RG and G6P found on SSC14 was adjacent to the *MINPP1* gene. The protein encoded by *MINPP1* is multiple inositol polyphosphate phosphatase, which can remove 3-phosphate from inositol phosphate substrates and convert 2,3 bisphosphoglycerate (2,3-BPG) to 2-phosphoglycerate that is part of the glycolytic pathway (Cho et al., 2008). The related pathways of *MINPP1* include Rapoport-Luebering glycolytic shunt and inositol phosphate metabolism (Cho et al., 2008). Thus, *MINPP1* could be regarded as strong candidate genes for RG and G6P. In addition, the peak locus rs321246758 in the intron of *SLC8A3* on SSC7 accounted 8.7% of the phenotypic variance in RG. The *SLC8A3* gene encodes a member of the sodium/calcium exchanger integral membrane protein family. Calcium plays a crucial role in muscle contraction and energy metabolism (Berridge et al., 2003), and SLC8A3 contributes to Ca^2+^ transport during excitation-contraction coupling in muscle. Its loss leads to muscle necrosis and abnormal Ca^2+^ homeostasis (Michel et al., 2014). Therefore, *SLC8A3* is a good candidate for RG.

The two GWAS peaks rs318410870 on SSC7 and rs318442172 on SSC16 were common to RG, LAT and GP. The SNP rs318410870 is an intronic variant of the *RIPOR2* gene. RIPOR2 can inhibit the activity of small GTPase RhoA and regulate myoblast differentiation (Yoon et al., 2007). RhoA plays an important role in actin cytoskeleton reorganization through RhoA-ROCKS signaling pathway (Ridley and Hall, 1992). Khue Ha Minh Duong et al. indicated that RhoA mediates glucose transport by regulating the vesicle trafficking machinery in an Akt-independent manner (Duong and Chun, 2019), and David Wu et al. also showed that activation of RhoA induces celluar glycolysis through translocation of glucose transporter GLUT3, which provides energy for cell contraction (Wu et al., 2021). Therefore, RIPOR2 may influence the process of glucose metabolism by inhibiting RhoA. Another GWAS locus affecting three traits, rs318442172, is an intronic variant in *LIFR* gene. LIFR protein, a member of the type I cytokine receptor family, binds to a converter subunit gp130 to form a receptor complex which mediates the role of the leukemia inhibitory factor (LIF) in cellular differentiation and proliferation (Hunt and White, 2016). Jessica C Hogan et al. showed that LIF regulates the expression of genes involved in lipid synthesis and has an impact on insulin-stimulated glucose uptake (Hogan and Stephens, 2005). In addition, Suhu Liu et al. found that LIFR can regulates the expression level of glucose transporter GLUT1, and the reduced expression level of LIFR leads to an increase in GLUT1 expression level, glycolysis and mitochondrial respiration (Liu et al., 2021). Thus, *LIFR* and *RIPOR2* could be regarded as candidate genes for the GP-related traits.

Two QTLs with the peak SNPs rs697205060 and rs345106152 were detected to affect both GP and LAT. The SNP rs697205060 is located in the intron of the *SRGAP1* gene, which encodes a GTPase activator for RhoA and Cdc42. As mentioned above, RhoA plays an important role in glucose transport (Ridley and Hall, 1992; Duong and Chun, 2019; Wu et al., 2021). Therefore, *SRGAP1* may influence the process of glucose metabolism by activating RhoA, and could be regarded as a promising candidate gene for the GP-related traits.

In addition to the pleiotropic loci, several possible candidate genes for the QTL associated with a single trait were also recognized. For example, the *FTO* gene containing the significant SNP rs338058884 for RG is a promising candidate. Melina Claussnitzer et al. found that the SNP rs1421085 in *FTO* gene disrupts a conserved motif for the *ARID5B* repressor, which leads to derepression of a potent preadipocyte enhancer and an increase in lipid storage with the increasing expression of IRX3 and IRX5 (Claussnitzer et al., 2015). Moreover, *FTO*’s related pathways include Glucose/Energy Metabolism and many studies have shown that *FTO* is closely related to glucose metabolism (Maia-Landim et al., 2018; Yang et al., 2019; Krüger et al., 2020; De Soysa et al., 2021). So, *FTO* is likely involved in regulating muscle glycogen content. On SSC17, the coding gene closest to the peak SNP rs322341359 for RG is *FOXA2* that encodes Forkhead Box A2. As a transcription activator, FOXA2 has a role in regulation of the expression of genes responsible for glucose homeostasis. Ping Wang et al. showed that FOXA2 restrains the proliferation of liver progenitor cells by decreasing PI3K/Akt/HK2-mediated glycolysis (Wang et al., 2020). Jennifer N Dines et al. reported that aberrant glucose homeostasis occurs in an individual with a missense variant in *FOXA2* (Dines et al., 2019). Therefore, FOXA2 are likely related to the SSC17 QTL effect on RG.

The most significant GWAS signal (rs320783325 with *p* = 9.81 × 10^−8^) for G6P was present in the *LIMCH1* gene. This gene encodes calponin homology domains-containing protein 1 which enables myosin II head/neck binding activity. The absence of LIMCH1 can affects the formation of actin stress fibers as well as the stability of focal adhesions, which are basic structures to ensure benign contraction and expansion in skeletal muscle (Lin et al., 2017). Fiuza-luces et al. showed that there was a significant relationship between LIMCH1 expression and adaptation of skeletal muscle to endurance training, independent of muscle glycogen availability (Fiuza-Luces et al., 2018). However, it is not ruled out that LIMCH1 alters the rate of glycolysis when it participates in stress pathways regulated by exercise, which warrants further investigation.

It is very important to identify genetic loci affecting GP phenotypes for genetic improvement of meat quality traits, as demonstrated by the application of mutations in *PRKAG3* and *PHKG1*, two known major genes in pig breeding (Galve et al., 2013). In this study, we identified five GP-associated peak SNPs, with minor allele frequencies of 0.127–0.434 (Table 2), indicating that there is sufficient room for selection of these loci in Erhualian pigs. More importantly, we observed that the genotype combinations of these five loci significantly affected not only GP, but also pH, color and water-holding capacity of meat (Figure 4). Therefore, the results provide a reference for improving meat quality uniformity and breeding efficiency of Erhualian pigs by multi-marker assistant selection.

## Conclusion

We found that the GP content in the skeletal muscle of Erhualian pigs was low and had a wide range of phenotypic variation, which may play a role in the formation of specific meat quality traits of this breed. Further, our GWAS analysisin the Erhualian population identified 31 loci significantly associated with the GP related traits, including 29 novel loci. Among them, six loci exhibited pleiotropic effects on these GP related traits. *SCL8A3*, *MINPP1*, *SRGAP1*, *RIPOR2*, and *LIFR* near the pleiotropic QTL peaks are highlighted as novel candidate genes related to glucose metabolism in muscle, which are worthy of further study. We also demonstrate the potential application of GP-associated SNPs in improving a variety of meat quality traits. Therefore, this study not only advances our understanding of the genetic architecture of GP related traits but also provide genetic markers for improving the pork quality of Erhualian.

## Data Availability

The data analyzed in this study is subject to the following licenses/restrictions: Datasets belong to Jiangxi Agricultural University. Requests to access these datasets should be directed to ma_junwu@hotmail.com.

## Supplementary material

SUPPLEMENTARY FIGURE S1 The Q-Q plots for 4 GP-related traits. The x axis represents the expected -log10 (P) and the y axis represents the observed -log10 (P)

SUPPLEMENTARY FIGURE S2 The PCA plots for the genotype data of Erhuanlian population

SUPPLEMENTARY FIGURE S3 The pleiotropic QTL for residual glycogen and glucose (RG) and glycolytic potential (GP) in muscle at about 93.78 Mb on SSC7. **(A)** Regional association plots of the QTL region of 4 Mb centered by the peak SNP (in red dots). Different colors stand for degrees of linkage disequilibrium (r2) between corresponding SNPs and the peak SNP. **(B)** Boxplots showing the difference in RG and GP values between the three genotypes of the common significant SNP rs322439157. **(C)** The physical locations of genes surrounding the significant SNPs
