# Supplementary material for: Genetic architecture for skeletal muscle glycolytic potential in Chinese Erhualian pigs revealed by a genome-wide association study using 1.4M SNP array
Source: Front Genet. 2023 Mar 17;14:1141411. doi: 10.3389/fgene.2023.1141411 (PMC10064215; doi:10.3389/fgene.2023.1141411)
Supplement: Supplementary file 1 [file Table1.DOCX]

Table S1. The genotype combinations of 5 GWAS peak SNPs for GP.

| Ord | Genotype combination^1^ | Allele number^2^ | Sample number^3^ | Group^4^ |
| --- | --- | --- | --- | --- |
| 1 | 11111 | 0 | 2 | A |
| 2 | 11112 | 1 | 3 | A |
| 3 | 11122 | 2 | 3 | A |
| 4 | 11131 | 2 | 1 | A |
| 5 | 12111 | 1 | 1 | A |
| 6 | 12121 | 2 | 1 | A |
| 7 | 21111 | 1 | 3 | A |
| 8 | 21112 | 2 | 12 | A |
| 9 | 21121 | 2 | 4 | A |
| 10 | 31111 | 2 | 1 | A |
| 11 | 11123 | 3 | 1 | B |
| 12 | 12122 | 3 | 2 | B |
| 13 | 12131 | 3 | 1 | B |
| 14 | 21113 | 3 | 1 | B |
| 15 | 21122 | 3 | 20 | B |
| 16 | 21123 | 4 | 7 | B |
| 17 | 21131 | 3 | 1 | B |
| 18 | 21132 | 4 | 7 | B |
| 19 | 21212 | 3 | 1 | B |
| 20 | 21221 | 3 | 1 | B |
| 21 | 21222 | 4 | 3 | B |
| 22 | 21223 | 5 | 2 | B |
| 23 | 21233 | 6 | 1 | B |
| 24 | 22112 | 3 | 7 | B |
| 25 | 22113 | 4 | 1 | B |
| 26 | 22121 | 3 | 3 | B |
| 27 | 22122 | 4 | 18 | B |
| 28 | 22123 | 5 | 5 | B |
| 29 | 22132 | 5 | 8 | B |
| 30 | 22222 | 5 | 1 | B |
| 31 | 22223 | 6 | 1 | B |
| 32 | 22233 | 7 | 1 | B |
| 33 | 23121 | 4 | 1 | B |
| 34 | 23122 | 5 | 2 | B |
| 35 | 23131 | 5 | 1 | B |
| 36 | 23222 | 6 | 1 | B |
| 37 | 23232 | 7 | 1 | B |
| 38 | 31112 | 3 | 2 | B |
| 39 | 31113 | 4 | 11 | B |
| 40 | 31122 | 4 | 12 | B |
| 41 | 31123 | 5 | 7 | B |
| 42 | 31132 | 5 | 7 | B |
| 43 | 31133 | 6 | 6 | B |
| 44 | 31212 | 4 | 1 | B |
| 45 | 31222 | 5 | 1 | B |
| 46 | 31223 | 6 | 4 | B |
| 47 | 31232 | 6 | 4 | B |
| 48 | 31233 | 7 | 3 | B |
| 49 | 32111 | 3 | 1 | B |
| 50 | 32112 | 4 | 2 | B |
| 51 | 32113 | 5 | 6 | B |
| 52 | 32122 | 5 | 6 | B |
| 53 | 32123 | 6 | 14 | B |
| 54 | 32131 | 5 | 1 | B |
| 55 | 32132 | 6 | 2 | B |
| 56 | 32133 | 7 | 15 | B |
| 57 | 32213 | 6 | 1 | B |
| 58 | 32222 | 6 | 5 | B |
| 59 | 32223 | 7 | 4 | B |
| 60 | 32232 | 7 | 5 | B |
| 61 | 32322 | 7 | 1 | B |
| 62 | 33113 | 6 | 1 | B |
| 63 | 33122 | 6 | 3 | B |
| 64 | 33123 | 7 | 6 | B |
| 65 | 33132 | 7 | 2 | B |
| 66 | 33222 | 7 | 2 | B |
| 67 | 23233 | 8 | 14 | C |
| 68 | 32323 | 8 | 1 | C |
| 69 | 32332 | 8 | 1 | C |
| 70 | 33133 | 8 | 4 | C |
| 71 | 33232 | 8 | 1 | C |
| 72 | 33233 | 9 | 6 | C |
| 73 | 33333 | 10 | 3 | C |

^1^ Genotype combination of top SNPs at five pleiotropic loci that affected GP and other components, and the top SNPs were rs318410870, rs318442172, rs697205060, rs345106152, rs332409349 in order. We classified each pair of alleles into 1,2,3 according to the number of GP enhancing alleles that each SNP contained. 1 contained a pair of alleles that both down-regulate GP, 2 contained an allele that enhances GP and an allele that down-regulates GP, and 3 contained a pair of alleles that both enhance GP.

^2^ The total number of alleles that each genotype combination contains to improve GP.

^3^ The number of individuals contained in each genotype combination.

^4^ The group to which each genotype combination belonged.
