# Supplementary figures and images for: Genetic architecture for skeletal muscle glycolytic potential in Chinese Erhualian pigs revealed by a genome-wide association study using 1.4M SNP array
Source: Front Genet. 2023 Mar 17;14:1141411. doi: 10.3389/fgene.2023.1141411 (PMC10064215; doi:10.3389/fgene.2023.1141411)

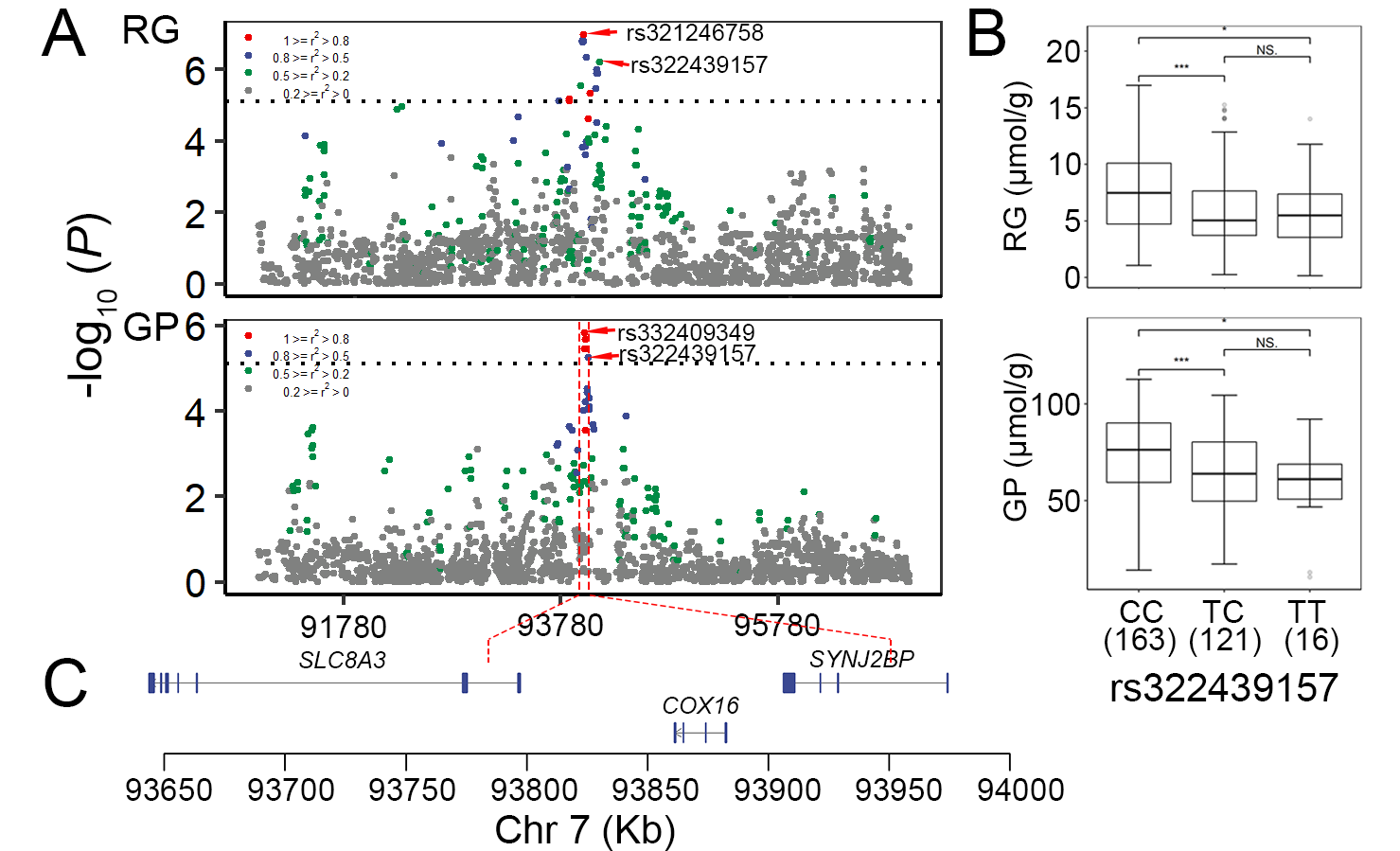

Supplement: Supplementary file 2 [file Image3.tif]

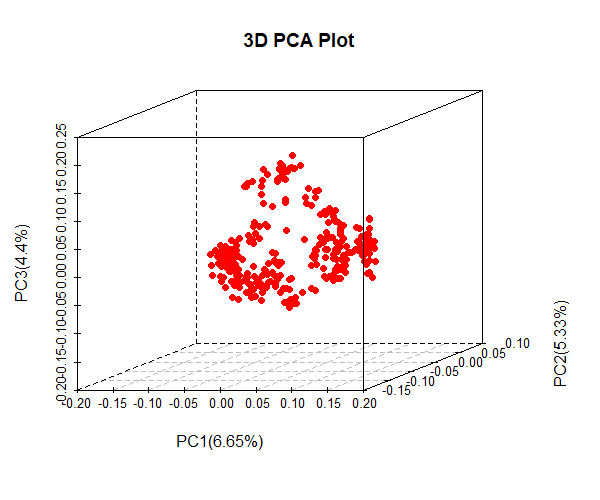

Supplement: Supplementary file 3 [file Image2.tif]

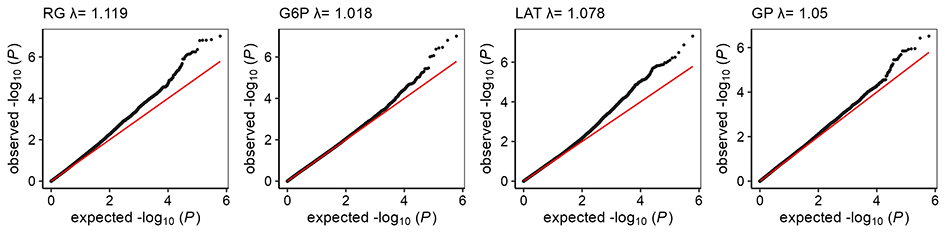

Supplement: Supplementary file 4 [file Image1.tif]
